# Advanced strain elastography is a reliable approach for prostate cancer detection in patients with elevated PSA levels

**DOI:** 10.1038/s41598-024-53440-2

**Published:** 2024-02-05

**Authors:** Yassir Edrees Almalki, Mohamed Gamal El-Din Mansour, Susan Adil Ali, Mohammad Abd Alkhalik Basha, Moustafa Mahmoud Abdelkawi, Sharifa Khalid Alduraibi, Ziyad A. Almushayti, Asim S. Aldhilan, Mervat Aboualkheir, Darine Amin, Mohamed Metkees, Ahmed M. A. Basha, Noha Yahia Ebaid

**Affiliations:** 1 Division of Radiology, Department of Internal Medicine, Medical College, Najran University, Najran, 61441 Kingdom of Saudi Arabia; 2 Department of Radiology, Faculty of Medicine, Ain Shams University, Cairo, 11566 Egypt; 3 Department of Radiology, Faculty of Medicine, Zagazig University, Zagazig, 44519 Egypt; 4 Department of Radiology, Faculty of Human Medicine, Helwan University, Cairo, 11795 Egypt; 5 Department of Radiology, College of Medicine, Qassim University, Buraidah, 52571 Kingdom of Saudi Arabia; 6 Department of Radiology and Medical Imaging, College of Medicine, Taibah University, Madinah, 42353 Kingdom of Saudi Arabia; 7 Department of Biological Anthropology, Medical Research Division, National Research Centre, Giza, 12622 Egypt; 8 Faculty of General Medicine, St. Petersburg State University, Egypt Branch, Cairo, 11646 Egypt

**Keywords:** Prostate, Cancer

## Abstract

This study aimed to examine the validity and reproducibility of strain elastography (SE) for detecting prostate cancer (PCa) in patients with elevated prostate-specific antigen (PSA) levels. The study included 107 patients with elevated PSA levels. All eligible patients underwent transrectal ultrasound (TRUS) with real-time elastography (RTE) to detect suspicious lesions. Two readers independently evaluated the lesions and assigned a strain ratio and elastography score to each lesion. Histopathology was used as a reference standard to estimate the validity of RTE in predicting malignant lesions. An intraclass correlation (ICC) was performed to detect reliability of the strain ratios and elastography scores. TRUS-guided biopsy detected malignancies in 64 (59.8%) patients. TRUS with RTE revealed 122 lesions. The strain ratio index (SRI) cut-off values to diagnose malignancy were 4.05 and 4.35, with sensitivity, specificity, and accuracy of 94.7%, 91.3%, and 93.4%, respectively. An elastography score > 3 was the best cut-off value for detecting malignancy. According to readers, the sensitivity, specificity, and accuracy were 91.3–94.7%, 89.5–93.4%, and 91.3–90.9%, respectively. Excellent inter-reader agreement was recorded for SRI and elastography scores, with ICC of 0.937 and 0.800, respectively. SE proves to be an efficient tool for detecting PCa with high accuracy in patients with elevated PSA levels.

## Introduction

Prostate cancer (PCa) ranks as the second leading cause of death in males, following lung cancer^1^. PCa is suspected when there are elevated serum prostatic specific antigen (PSA) levels or a positive digital rectal examination (DRE) result, and it is confirmed via histopathological examination through transrectal ultrasound (TRUS)-guided systematic biopsy. Gray scale ultrasound only reveals PCa in 9–53% of suspicious hypoechoic areas. Owing to its low cost and widespread availability, this method is considered the gold standard for PCa detection. However, diagnosis is often missed in many patients^2–4^. Even when PCa is detected, it may be understaged if the biopsy misses the most aggressive area of the lesion^5^. Moreover, this method can lead to overdiagnosis by identifying clinically insignificant (low-grade) cancers, potentially resulting in expensive overtreatment that negatively impacts the patient's quality of life^4,6^.

Real-time elastography (RTE), a recent innovation in prostate imaging, is a non-invasive and cost-effective tool that visualizes PCa with high sensitivity^7^. The foundation of RTE under real-time conditions is the significant difference in stiffness between the neoplastic and normal prostate tissues. Currently, two different techniques, strain elastography (SE) and shear wave elastography (SWE), are used to demonstrate prostatic tissue elasticity. Among these, SE is the most widely used technique for assessing prostate tissue stiffness^8^.

This study evaluated the efficacy of SE in detecting PCa in patients with elevated PSA levels who were referred for TRUS biopsy, which served as the reference standard. The reliability of the SE and level of inter-reader agreement were also assessed.

## Materials and methods

We adhered to the Standards for Reporting Diagnostic Accuracy (STARD) statement guidelines for conducting this diagnostic test accuracy (DTA) study. The study received approval from the Institutional Review Board (reference number: REC-FMHU 92–2022), and a written informed consent was obtained from the patients or their legal guardians for publication of the accompanying images. The study was conducted in accordance with the ethical principles outlined in the Declaration of Helsinki.

### Patients

**Figure 1:**
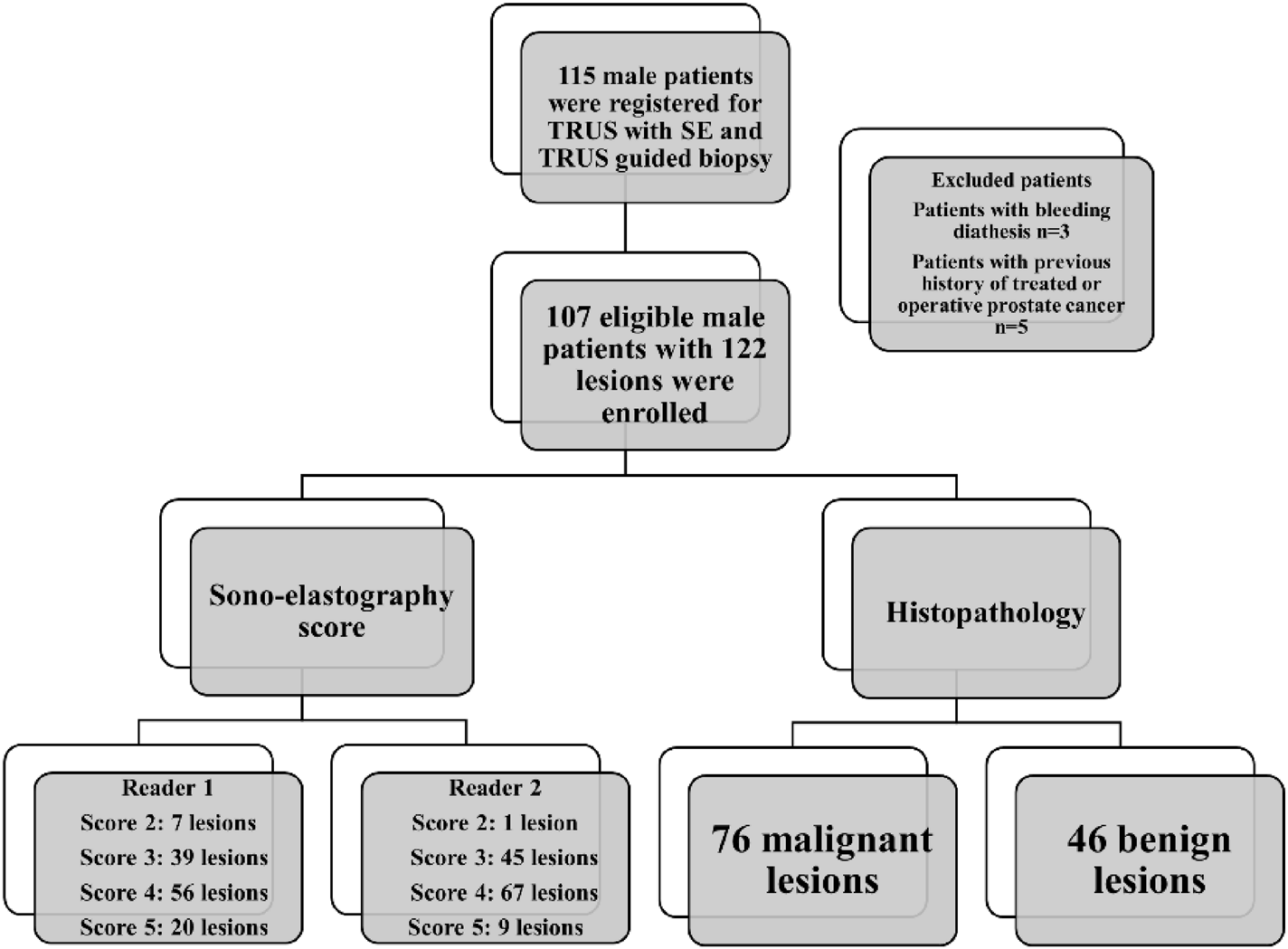
Flow chart of the study process.

This single-center DTA study was conducted from September 2022 to March 2023. A total of 115 consecutive patients were enrolled for TRUS with SE and TRUS-guided biopsies. The inclusion criteria stipulated patients with elevated PSA serum levels who were referred for TRUS with SE, followed by TRUS-guided biopsies. We excluded patients with bleeding diathesis (n = 3) and those with a history of treated or operated PCa (n = 5). The final sample consisted of 107 eligible male patients. The flowchart of the study process is shown in Fig. 1.

### TRUS and SE imaging technique

#### Patient preparation

All patients were instructed to discontinue the use of anticoagulants 5–7 days prior to the procedure. They underwent enemas 24 h and 6 h before the examination. Prophylactic oral antibiotics were initiated one day prior to the procedure and continued for a duration of five days.

#### Prostate strain elastography technique

A Samsung Premium Ultrasound System (RS80A with Prestige) equipped with an E3-12A ultrasound transducer was used for examination. Patients were positioned on their left side. The transducer, coated with a coupling gel, was gently inserted into the rectum. Initially, B-mode ultrasonography was employed to depict anatomy. The entire prostate, from apex to base, was scanned in both the sagittal and transverse planes to identify zonal anatomy and measure prostatic volume. The outer peripheral zone of a normal gland appears homogeneous and mildly hyperechoic compared with the inner transitional or central zones. Hypoechoic soft tissue lesions were suspected to be PCa. Continuous manual mild compressions and decompressions were then applied with a transrectal probe; the elastography box encompassed the prostate and surrounding tissues while avoiding the urinary bladder. A circle was drawn around the region of interest (ROI) (2–5 mm) in both the normal and suspicious prostatic areas to assess tissue stiffness and measure the strain ratio.

### Image analysis

The elastogram is a color map that overlies the strain values in the B-mode image. Blue indicates low-strain (stiff) tissues, whereas red indicates high-strain (soft) tissues (although color scales may vary by manufacturer). Typically, the peripheral prostatic zone exhibits an intermediate stiffness. In contrast, the central and transitional zones (central gland) display a homogeneous soft pattern in young males, with stiffness and volume increasing with age. Moreover, the prostatic capsule normally has a pericapsular soft rim artifact, which is lost with the extracapsular extension of PCa. Two readers, each with five years of experience in uroradiology, independently assessed the prostatic lesions using SE. A 5-point scoring system (1–5) was used for semi-quantitative assessment of the elastogram data, with the highest scores indicating a high probability of malignancy. The strain ratio index (SRI), an alternative semi-quantitative measurement, is the ratio of the peak strain in normal prostatic tissue to that in suspicious lesions. This ratio was significantly higher in malignant lesions than in benign lesions.

### The technique of prostatic biopsy (gold standard)

TRUS-guided 12 cores biopsies were performed in all patients. In addition, two other biopsy cores were obtained for each suspicious lesion detected on the sonoelastographic images. All biopsies were performed by an experienced uroradiologist under periprostatic nerve block using 7 cc of 1% lidocaine injected via a spinal needle just lateral to the junction between the prostatic base and seminal vesicles, bilaterally. Two-uropathologists with over 10 years of experience checked all the prostatic specimens.

### Statistical analysis

Statistical analyses were performed using the IBM SPSS Statistics software (version 24.0, IBM Corp., USA, 2016). The Kolmogorov–Smirnov test was used to assess the data distribution. Continuous data are presented as mean ± standard deviation (SD) or median and interquartile range (IQR), as appropriate. Qualitative data are represented as the number (n) and percentage (%). To assess the diagnostic validity of SE, a receiver operating characteristic (ROC) analysis was performed, considering the histopathological results as the gold standard. This analysis provided the maximum accuracy points for both sensitivity and specificity. Additionally, the area under the curve (AUC), negative predictive value (NPV), and positive predictive value (PPV) were determined. The intraclass correlation coefficient (ICC) was used to assess the reliability of SE with respect to the SRI and elastography scores of the two readers. ICC values range between 0 and 1, with values less than 0.50, between 0.50 and 0.75, between 0.75 and 0.90, and greater than 0.90 indicating poor, moderate, good, and excellent agreement, respectively. P value < 0.05 was considered statistically significant.

### Institutional review board statement

This study was approved by the Institutional Review Board (Approval No: REC-FMHU 92-2022, approved on October 02, 2022).

### Informed consent statement

Informed consent was obtained from each patient.

## Results

### Demographic and basic characteristics

The study involved 107 males with elevated serum PSA levels who were referred for TRUS with SE and TRUS-guided biopsy. The median age (IQR) was 62 (11) years, and the median serum PSA level (IQR) was 14 (10) ng/ml. Histopathology identified 76 malignant and 46 benign lesions. The most prevalent Gleason pattern was 3 + 4, found in 22 (34.4%) patients. The basic and clinical characteristics of the patients are summarized in Table 1.

**Table 1:** Patients’ basic characteristics in the study group.

Variables		Study group (n = 107)	
		No	(%)
Number	Patients	107	
	Lesions	122	
Age (year)	Median (IQR)	62 (11)	
PSA ng/ml	Median (IQR)	14 (10)	
Gleason Pattern	3 + 3	12	18.8
	3 + 4	22	34.4
	4 + 3	16	25.0
	8	8	12.5
	9	4	6.3
	10	2	3.1
Histopathology	Benign per patient	43	40.2
	Malignant per patient	64	59.8
	Benign per lesion	46	37.7
	Malignant per lesion	76	62.3

*IQR* interquartile range.

### Frequency distribution of SE results between the two readers

The frequency distribution of different SE items, as determined by the two readers, is provided in Table 2. SE identified 76 suspicious lesions (scores 4 and 5) by both readers. These lesions were located in the peripheral zone, the transitional zone, or both. The SRI median (IQR) were 4.9 (3.00) and 4.9 (3.05) for both readers. According to readers, the most prevalent elastography score was 4 in 56 and 67 lesions, respectively.

**Table 2:** Frequency distribution of different items of SE by the two readers.

Characteristic	Reader 1	Reader 2
SRI, Median (IQR)	4.9 (3.00)	4.9 (3.05)
Elastography Score		
2	7 (5.7)	1 (0.8)
3	39 (32.0)	45 (36.9)
4	56 (45.9)	67 (54.9)
5	20 (16.4)	9 (7.4)

Unless otherwise indicated, the data are presented as numbers and percentages in parentheses.

*SRI* strain ratio index, *ICR* interquartile range.

### Diagnostic performance of SE in detecting malignant prostatic lesions

The diagnostic accuracy of SRI on lesion-based analysis is outlined in Table 3. Suspiciously stiff lesions predominantly appeared blue on elastographic map (scores 4 and 5). The SRI cut-off values for diagnosing malignancy were 4.05 and 4.35. These provided a sensitivity, specificity, and accuracy of 94.7%, 91.3%, and 93.4%, respectively (P < 0.001). Regarding the elastography score, a score > 3 was determined as the best cut-off value for detecting malignancy. This resulted in a sensitivity, specificity, and accuracy of 89.5%, 91.3%, and 90.9%, and 94.7%, 91.3%, and 93.4% for the two readers, respectively (P < 0.001) (Table 4).

**Table 3:** The validity of the SRI using histopathology as the gold standard.

	Reader 1	Reader 2
AUC	0.936	0.924
Cut off	> 4.35	> 4.05
CI	0.886–0.986	0.869–0.980
Sensitivity	94.7%	94.7%
Specificity	91.3%	91.3%
PPV	94.7%	94.7%
NPP	91.3%	91.3%
Accuracy	93.4%	93.4%

*SRI* strain ratio index, *AUC* area under the curve, *CI* confidence interval, *PPV* positive predictive value, *NPP* negative predictive value.

**Table 4:** The validity of the elastography score using histopathology as the gold standard.

	Reader 1	Reader 2
AUC	0.932	0.903
Cut off	> 3	> 3
CI	0.881–0.984	0.842–0.964
Sensitivity	94.7%	89.5%
Specificity	91.3%	91.3%
PPV	94.7%	94.4%
NPV	91.3%	84%
Accuracy	93.4%	90.9%

*AUC* area under the curve, *CI* confidence interval, *PPV* positive predictive value, *NPP* negative predictive value.

### ROC curves analyses

**Figure 2:**
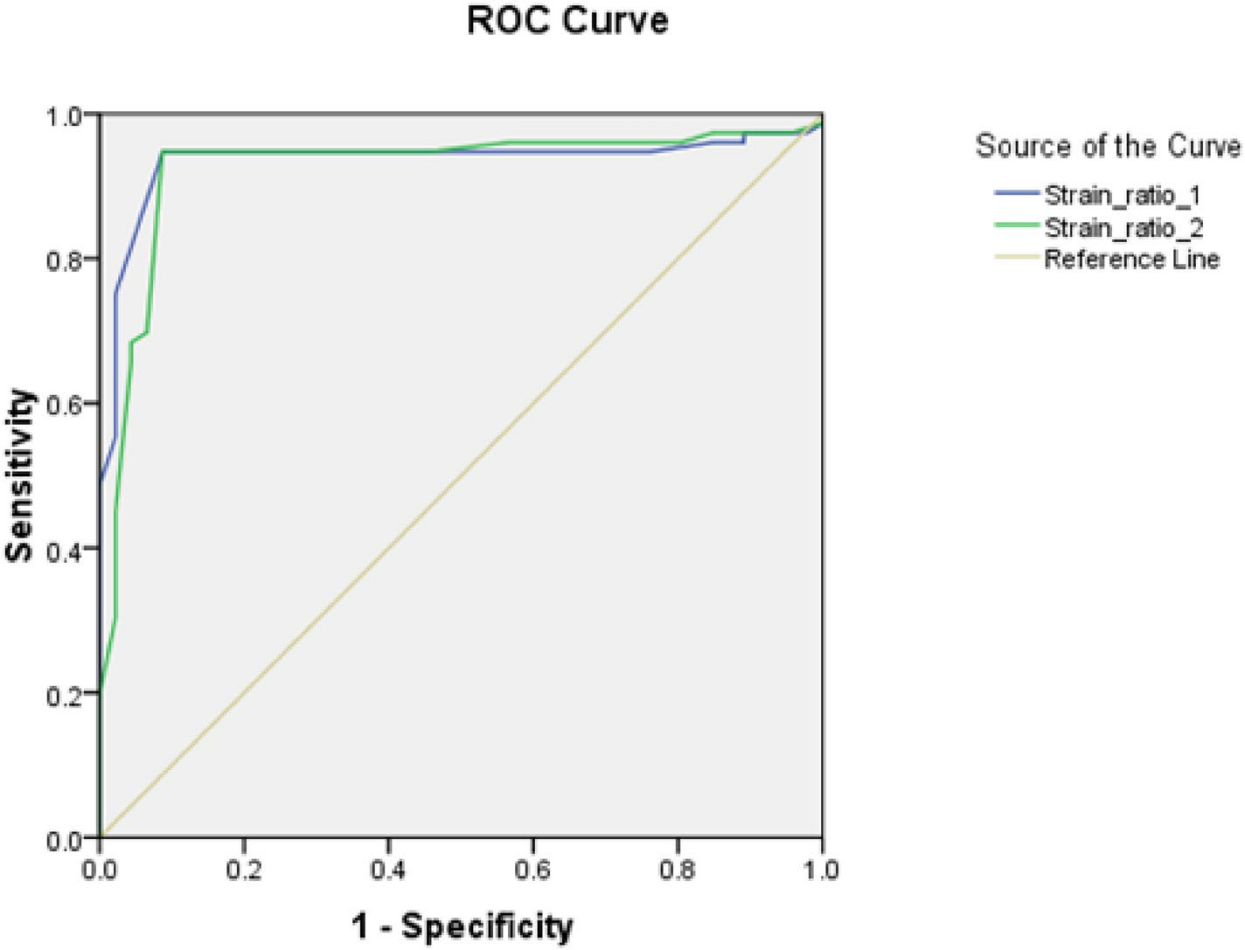
ROC curve analysis of the strain ratio cut-off for both readers.

**Figure 3:**
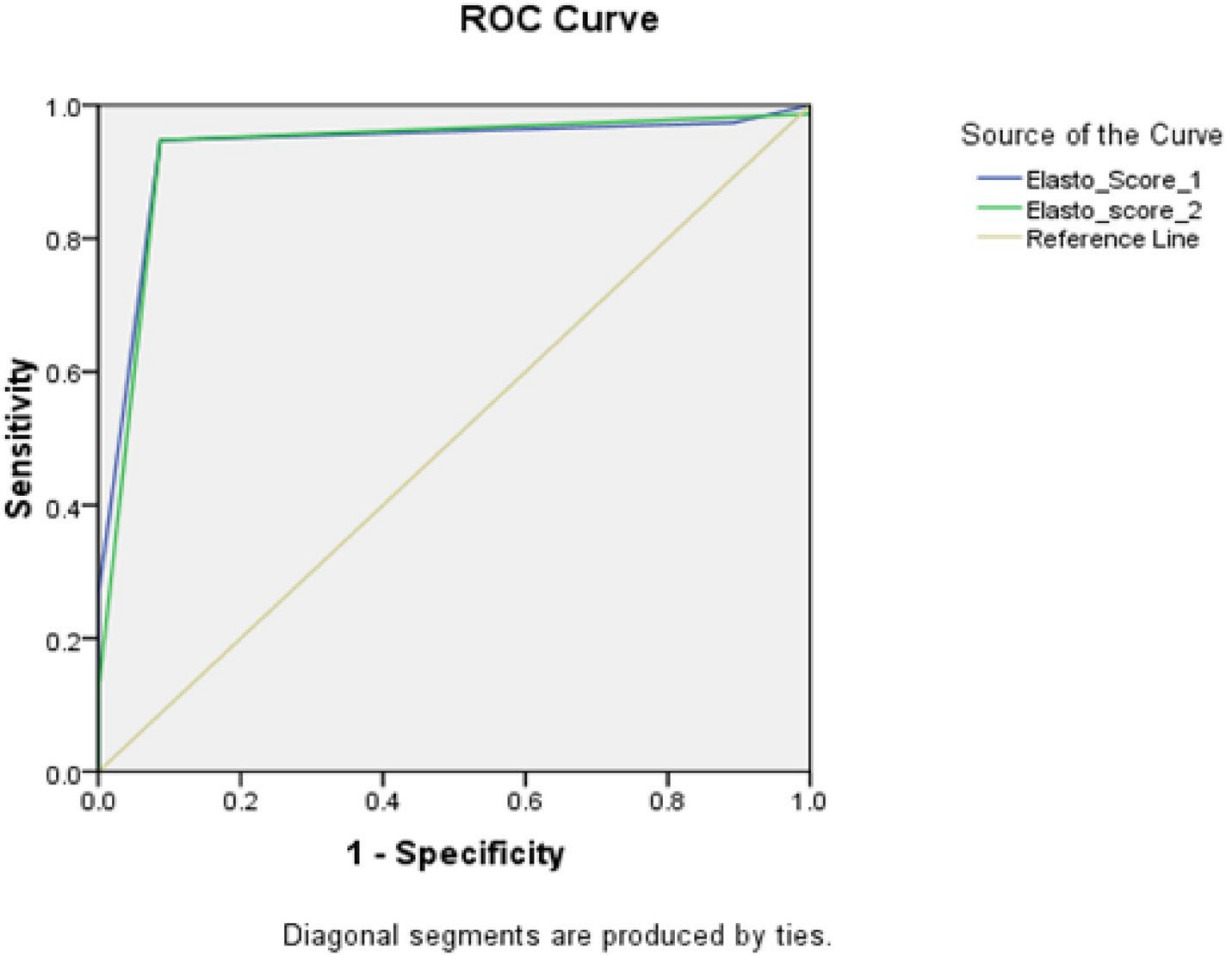
ROC curve analysis of the elastography score cut-off for both readers.

The ROC curve was used to estimate the optimal cutoff values of the SRI and elastography score for both readers. Depending on the readers, SRI cutoff of 4.05 and 4.35 was determined to diagnose malignancy, with an AUC of 0.924 (95% CI, 0.869–0.980) and 0.936 (95% CI, 0.886–0.986), respectively (Fig. 2). An elastography score greater than 3 was recorded as the best cut-off value to predict malignancy. This score resulted in an AUC of 0.903 (95% CI x, 0.842–0.964) and 0.932 (95% CI, 0.881–0.984) for the two readers, respectively (Fig. 3).

### Inter-reader reliability of SE parameters

The inter-reader agreement of the SE parameters is summarized in Table 5. The overall reliability of the SE parameters was excellent, with SRI and elastography scores of 0.937 and 0.800, respectively.

**Table 5:** Inter-reader reliability of the SE parameters.

Characteristic	ICC (95% CI)	Cronbach’s Alpha	P value
Strain ratio	0.937 (0.911–0.956)	0.968	< 0.001
Elastography score	0.800 (0.726–0.856)	0.889	< 0.001

*ICC* intraclass correlation coefficient, *CI* confidence interval.

### Association between the elastography scores of both readers and histopathological results

Table 6 demonstrates a statistically significant association between elastography scores of both readers and the histopathological result, with malignant lesions having high elastography scores (P < 0.001).

**Table 6:** Association between elastography score and histopathological results.

Elastography score	Reader 1		Reader 2		P-value
	Benign	Malignant	Benign	Malignant	
Score 2	5	2	0	1	< 0.001*
Score 3	37	2	42	3	
Score 4	4	52	4	63	
Score 5	0	20	0	9	

*, Fisher exact test; P < 0.05, considered statistically significant.

**Figure 4:**
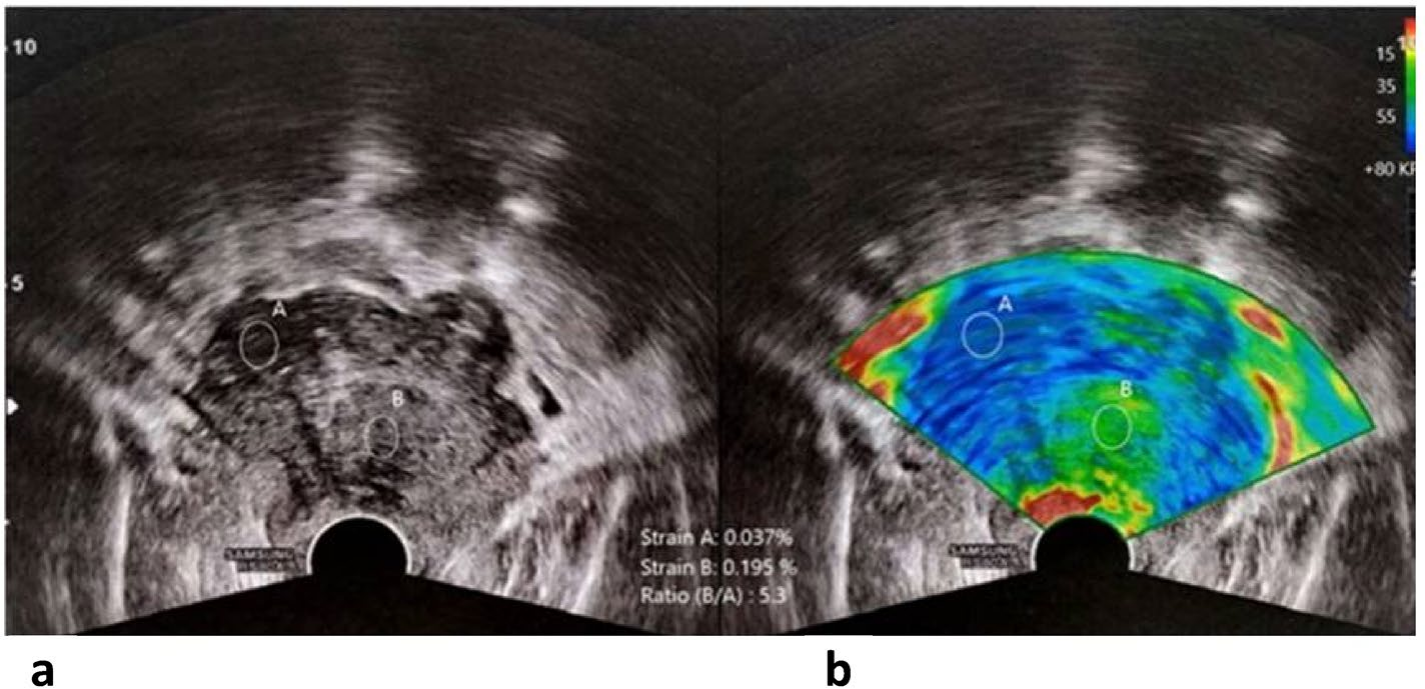
A 66-year-old male presented with a high serum PSA level. The B-mode image (**A**) shows an inhomogeneous hypoechoic texture of most of the gland, and the color-coded SE (**B**) at the same plane displays the blue color of the suspicious prostate parenchyma, especially its right aspect (suggesting firm consistency and low elasticity). Two rounded regions of interest (ROI) were placed, and the strain ratio was measured between the relatively soft area (green) and the suspicious right transitional zone area (blue), which was 5.3 (high ratio), confirming its firm consistency. Biopsies were obtained from the suspicious prostatic regions and revealed Gleason 9 prostatic adenocarcinoma.

**Figure 5:**
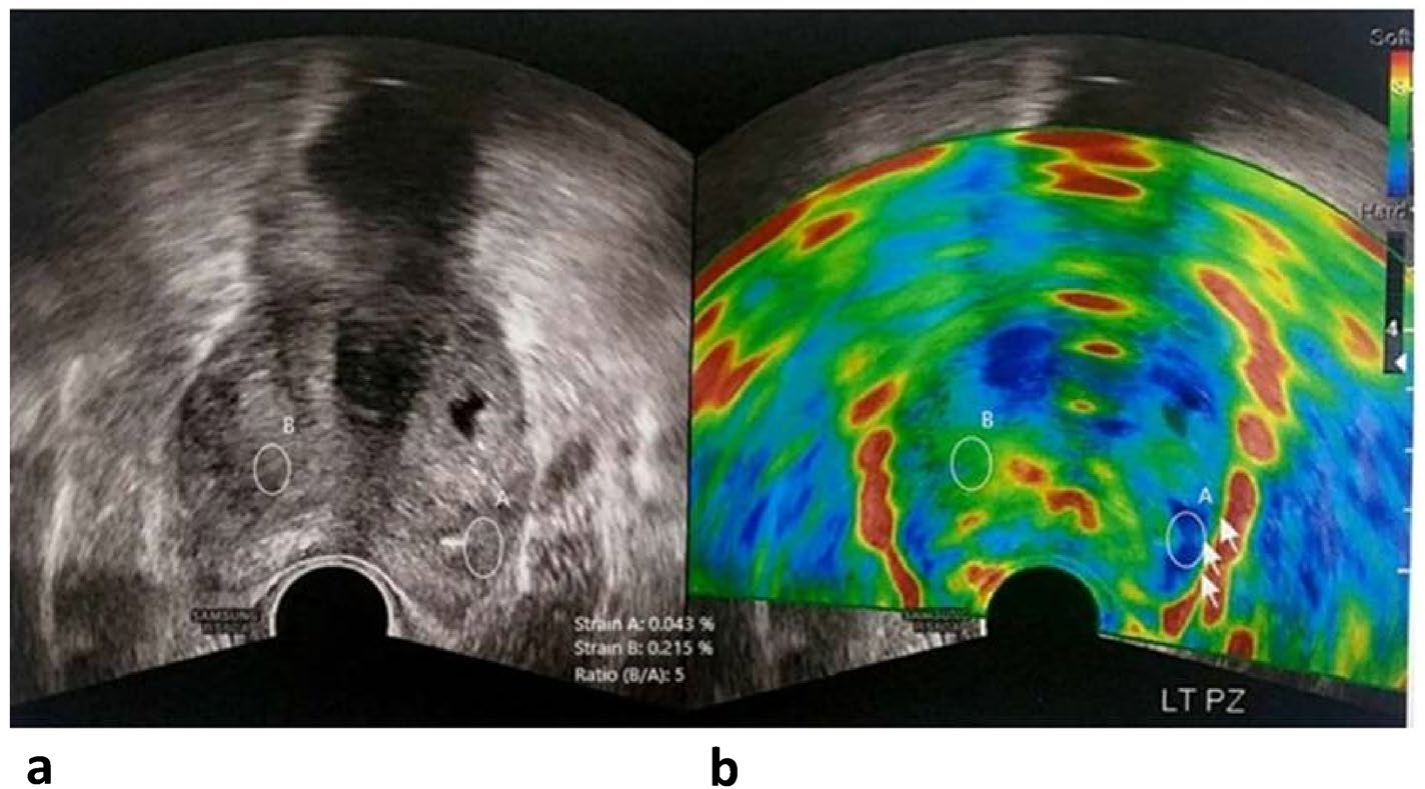
A 71-year-old male presented with markedly elevated serum PSA. The B-mode image (**A**) shows a suspicious hypoechoic inhomogeneous texture in the left and right peripheral zones, as well as the transitional zone displaying blue color in the color-coded SE (**B**), suggesting firm consistency and low elasticity. Two rounded regions of interest (ROI) were placed, and the strain ratio was measured between the relatively soft area (green) and the suspicious (blue) left peripheral zone area, which was 5 (high ratio), confirming its firm consistency. Biopsies were performed, which revealed Gleason 9 PCa.

**Figure 6:**
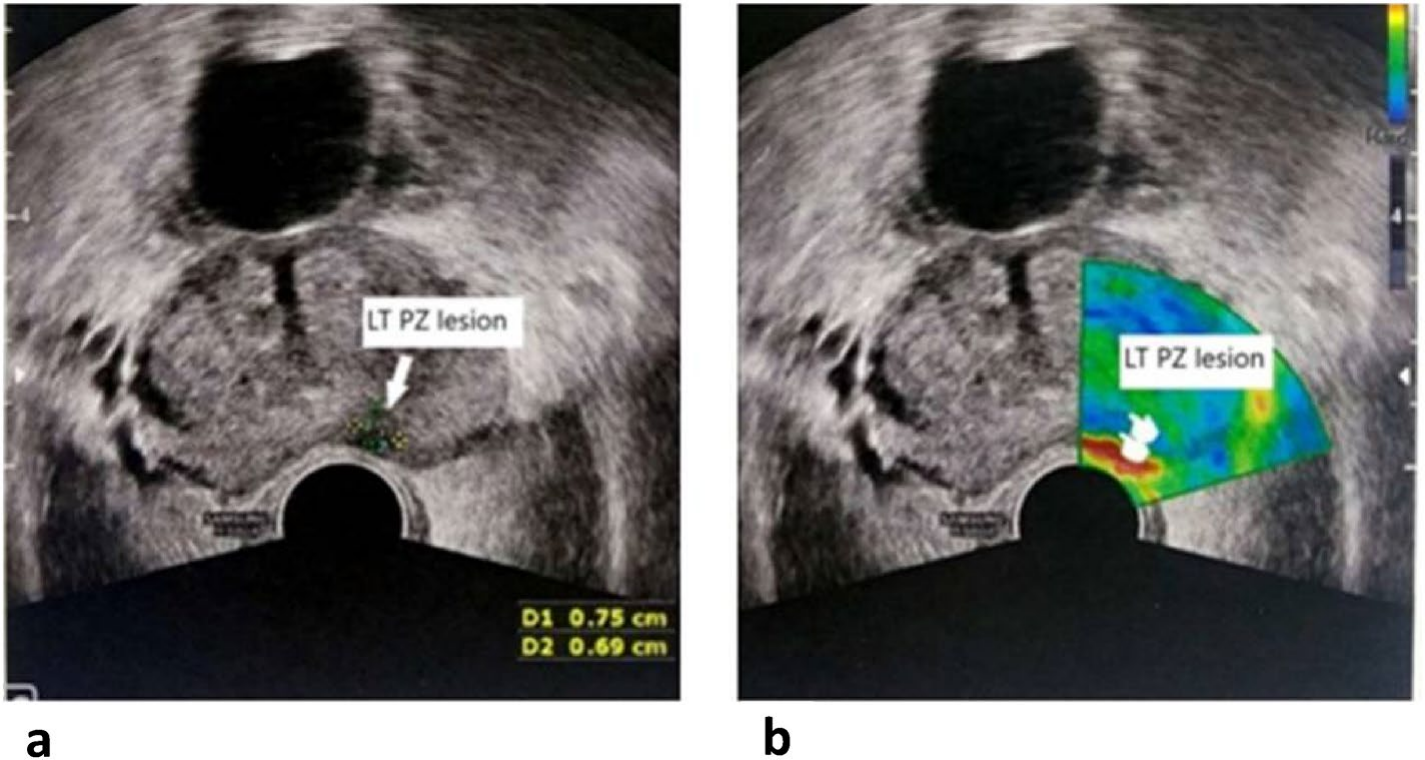
A 57-year-old male presented with a high serum PSA level. The B-mode image (**A**) shows a small paramedian hypoechoic lesion in the left peripheral zone, displaying a blue color on color-coded SE (**B**), suggesting firm consistency and low elasticity. Biopsies revealed a Gleason 7 prostatic adenocarcinoma.

Our representative cases are demonstrated in (Figs. 4, 5, 6).

## Discussion

The current study demonstrated high diagnostic performance of SE in detecting PCa. Furthermore, excellent agreement was observed between the readers concerning the overall SE results, particularly in relation to the SRI and elastography scores. A statistically significant association was reported between elastography scores of both readers and histopathological findings. This study extends the current literature by providing information on the cut-off values for the SRI and elastography score when predicting PCa. This information is particularly relevant for patients with suspicious lesions and elevated PSA levels.

In a study by Ferrari et al.^9^, 84 patients with suspected PCa underwent TRUS with SE and transperineal prostate biopsy. The sensitivity, specificity, PPV, and NPV of SE were reported to be 66%, 78%, 77%, and 67%, respectively. However, the current study recorded higher diagnostic accuracy parameters, with sensitivity, specificity, and accuracy of 94.7%, 91.3%, and 93.4%, respectively, as determined by both readers.

Many studies^10–13^ have investigated suspicious prostatic lesions in patients with elevated PSA levels to determine the optimal cut-off value of the SRI for diagnosing malignancy. However, these studies reported varying SRI values. This heterogeneity in SRI values may be attributed to the unavailability of stable reference prostatic tissue on the contralateral side of the gland, which might be affected by infection or tumor microinfiltration^14^. Other factors contributing to the heterogeneity of the strain ratio cut-off value might be the fact that SE is operator-dependent and can vary based on different examiners' expertise. The highest SRI was recorded by Zhang et al.^10^, This can be attributed to Zhang et al.'s usage of two methods of image analysis due to variable stiffness throughout the lesion. They calculated both the average strain index (SI), which is the strain ratio of the reference prostatic tissue to the strain ratio of the total lesion, and peak SI, which is the strain ratio of the reference prostatic tissue to the peak strain ratio. Furthermore, they reported in their study that some artifacts were erroneously calculated as positive regions, thereby increasing the cut-off value of the SI.

Regarding the optimal elastography score cut-off for diagnosing malignancy, the current study's cut-off of > 3 aligns with the study by Mahajan et al.^15^. Their research reported higher elastography scores in malignant lesions, achieving an accuracy of 92%, sensitivity of 85.7%, and specificity of 94.4%. Two previous studies^16,17^ reported the same elastography score cut-off value but exhibited lower diagnostic performance than the current study. These two studies reported sensitivities of 68% and 68.6%, specificities of 81% and 69.4%, and accuracies of 76% and 69.2%, respectively. These results could be attributed to the different machines used in each study and the fact that TRUS-SE is operator-dependent, leading to potential variations in results between examiners. However, TRUS-SE exhibited high inter-examiner reliability regarding the SRI and elastography scores. Consequently, we recommend conducting further prospective, multicenter studies to validate our findings. Moreover, the prevalence of PCa in the study by Xu et al.^17^ was lower than that in the current study (26.2% vs. 59.8%).

Regarding inter-reader agreement, the results of the current study are promising, showing excellent agreement between the readers in terms of the SRI and elastography score (ICC = 0.937 and 0.800, respectively). However, test–retest reliability was assessed between highly experienced readers. Consequently, we recommend further studies to evaluate the diagnostic accuracy of SE conducted by less experienced readers.

The current study reported a statistically significant association between elastography scores assessed by both readers and histopathology results. Benign lesions had lower elastography scores, whereas malignant lesions had higher scores. These findings align with those of Kanagaraju et al.^18^, who reported similar results in their study. This correlation can be attributed to the fact that malignant lesions are characterized by replacing glandular tissue with malignant cells, resulting in a higher stiffness than benign lesions.

SE boasts numerous strengths and may be the preferred choice by many urologists and uroradiologists. Several studies have compared SE-targeted biopsy and systematic biopsy, demonstrating that including SE improves PCa detection. For instance, a prospective study involving 353 patients unveiled superior PCa detection with SE-targeted biopsy (51.1%) compared with systematic biopsy (39.4%)^19^. Similarly, a prospective study conducted on 230 patients showed an improvement in PCa detection with SE-guided biopsy (30%) compared with systematic biopsy (25%)^20^. Furthermore, a large retrospective study involving 1024 patients reported a PCa detection rate of 24.8% after adding SE-guided biopsy to systematic biopsy in patients undergoing re-biopsy^21^. Moreover, Wang et al.^22^ reported a 13.9% increase in PCa detection after incorporating SE guidance. On the other hand, a retrospective study reported a low sensitivity (19.8%) but high specificity (90.9%) for SE-guided biopsy among 519 patients undergoing re-biopsy^23^. The detection of PCa using SE also depends on the tumor grade. High-grade tumors, characterized by increased cellular packing and stiffness, tend to improve SE sensitivity. Sumara et al.^24^ conducted an evaluation of elastography and reported PCa detection rates of 60%, 69.2%, and 100% for Gleason scores of 6, 7, and 8–9, respectively.

MRI has demonstrated outstanding results in the detection and localization of various tumors. Multiparametric (mp)-MRI, in conjunction with T2-weighted and diffusion-weighted imaging, plays a significant role in PCa detection^25^. The reported AUC exceeds 0.9^26^.The diagnostic accuracy of MRI varies because it depends on the combination of various features from T2-weighted images, diffusion-weighted images, dynamic contrast-enhanced images, and MR spectroscopy images in specific settings^27^. In addition, despite MRI's high sensitivity in detecting PCa, it exhibits low specificity due to increased vascularity in the inner gland and benign nodules, such as benign prostatic hyperplasia. Moreover, MRI sensitivity is low for detecting small malignant lesions with low Gleason scores^28^ and cannot differentiate between indolent and aggressive lesions^29^. Furthermore, when the results from different MRI sequences are inconsistent, radiologists find it challenging to combine such findings, impacting the reliability of MRI image analysis and interpretation between radiologists. Therefore, in low-resource settings, SE could be used to predict PCa with high diagnostic accuracy. If MRI is available, it is advantageous to couple it with SE to confirm the diagnosis, thereby helping reduce unnecessary biopsies.

The utilization of SE for PCa detection has a few limitations. First, it is a somewhat qualitative technique that presumes uniform prostate compression^30^. Using computational methods to reconstruct images, considering the non-uniformity of stress, may enhance the results^31^. Another limitation is the variability of the SE technique, which is operator-dependent and has a recognized learning curve^32^. Furthermore, elastogram color-code schemes depend on the ultrasound machine vendor and are not standardized^33^. Other factors, such as tumor location, size, and prostate volume, may influence performance. PCa detection by SE improves with increasing tumor size (9.7% in 0–5 mm lesions, 27% in 6–10 mm lesions, 70.6% in 11–20 mm lesions, and 100% in lesions > 20 mm)^34^. SE exhibits higher sensitivity at the prostatic apex (60–76.9%) than at the base (34.2–45%), primarily due to the better application of compression and decompression of the smaller volume at the apex^18^. Similarly, PCa detection rates are higher for smaller-volume prostates than large ones^17^. Additionally, false-positive results may occur in benign conditions, such as prostatic calcifications, fibrosis, prostatitis, adenomyomatosis, atrophy, and BPH^35^.

Finally, the appropriate and early diagnosis of PCa in patients suspected to have elevated PSA levels can be facilitated by non-invasive radiological investigations, such as SE, coupled with clinical and SE-guided pathological evaluations. This approach is suggested to reduce mortality rates and enhance survival prospects. However, we recommend further multicenter studies with larger sample sizes to establish and validate the optimal strain ratio cut-off for predicting malignancy. It is also important to correlate these results with multiparametric MRI and histopathology, both considered the gold standards for PCa diagnosis.

The study had some limitations. First, it was a single-center study, indicating the need for prospective multicenter studies to generalize results. Second, the high prevalence of malignant lesions may have skewed the accuracy of the parameter calculation. Third, mp-MRI was not used for comparison with SE. In a clinical setting, confirming the diagnosis and reducing unnecessary biopsies using SE in conjunction with mp-MRI is recommended. However, as previously mentioned, SE can be a valuable, non-invasive, rapid tool in low- and middle-income countries or low-resource settings where mp-MRI might be unavailable. With highly experienced operators, SE can predict malignant lesions with a higher diagnostic performance.

## Conclusions

TRUS-SE is a promising imaging tool for detecting cancerous tissues with high accuracy. Incorporating strain elastography-targeted biopsy into a systematic biopsy can enhance the detection rate of PCa in patients with elevated serum PSA levels.

## Data Availability

The datasets used and/or analyzed during the current study are available from the corresponding author upon reasonable request.
